# Mucosal Defense Against *Giardia* at the Intestinal Epithelial Cell Interface

**DOI:** 10.3389/fimmu.2022.817468

**Published:** 2022-02-17

**Authors:** Shahram Solaymani-Mohammadi

**Affiliations:** Laboratory of Mucosal Immunology, Department of Biomedical Sciences, School of Medicine and Health Sciences, University of North Dakota, Grand Forks, ND, United States

**Keywords:** giardiasis, *Giardia duodenalis*, mucosal immunity, intestinal barrier, epithelium, antimicrobial peptides, disaccharidase deficiency

## Abstract

Human giardiasis, caused by the protozoan parasite *Giardia duodenalis* (syn. *Giardia lamblia*, *Giardia intestinalis*, *Lamblia intestinalis*), is one of the most commonly-identified parasitic diseases worldwide. Chronic *G. duodenalis* infections cause a malabsorption syndrome that may lead to failure to thrive and/or stunted growth, especially in children in developing countries. Understanding the parasite/epithelial cell crosstalk at the mucosal surfaces of the small intestine during human giardiasis may provide novel insights into the mechanisms underlying the parasite-induced immunopathology and epithelial tissue damage, leading to malnutrition. Efforts to identify new targets for intervening in the development of intestinal immunopathology and the progression to malnutrition are critical. Translating these findings into a clinical setting will require analysis of these pathways in cells and tissues from humans and clinical trials could be devised to determine whether interfering with unwanted mucosal immune responses developed during human giardiasis provide better therapeutic benefits and clinical outcomes for *G. duodenalis* infections in humans.

## Introduction

Human giardiasis, caused by the protozoan parasite *Giardia duodenalis* (syn. *Giardia lamblia*, *Giardia intestinalis*, *Lamblia intestinalis*), is one of the most prevalent enteric parasitic protozoan infections globally, with prevalence rates ranging from 2-5% in the developed world and 20-30% in the developing countries (1–3). Infections with *G. duodenalis* account for more than 280 million of new cases of human giardiasis annually worldwide (4, 5). Epidemiological and molecular studies have classified *G. duodenalis* parasites into eight distinct and genetically-different parasites or “assemblages” (A-H) of which only assemblages A and B are typically identified in both humans and in other mammalian hosts, whereas assemblage E, for example, is predominantly identified in the livestock (6, 7). In recent years, however, assemblage E has also been identified to infect humans in Brazil (8, 9), Egypt (10), Australia (11), Vietnam (12) and New Zealand (13). This further indicates the potential for more widespread anthropozoonotic importance of *G. duodenalis* parasites and the roles played by numerous mammalian species in the maintenance of the parasite’s life cycle. The parasite’s life cycle follows a direct oral-fecal transmission route, and human infections are initiated by the ingestion of quadrinucleate cysts along with contaminated food or water (1). The ingestion of as few as 10-25 cysts would be enough to successfully colonize the small intestine (14). The vegetative forms of the parasite or trophozoites are binuclear pear-shaped flagellated structures with a bilateral symmetry that colonize the proximal portions of the small intestine, especially the duodenum and less commonly jejunum and the ileum (15).

Most cases of human giardiasis in immunocompetent individuals are self-limiting and and are spontaneously resolved within weeks following exposure (14). Individuals residing in hyperendemic areas for human giardiasis develop partial immunity against subsequent infections as opposed to newly arrived visitors (16, 17). These findings indicate the development of an effective anti-*Giardia* immunity sufficient for the clearance of *G. duodenalis* infections in humans. The majority of human giardiasis cases are asymptomatic with no signs of overt clinical profiles (18, 19). Human cases with asymptomatic giardiasis predominantly excrete infective cysts in the feces and play important roles in the maintenance of the parasite’s life cycle (1). Nonetheless, human subjects with symptomatic giardiasis mostly shed trophozoites in feces and are commonly presented with gastrointestinal manifestations that may include abdominal cramps, flatulence, diarrhea, nausea, with a malabsorption syndrome occurring in clinical and subclinical cases and may result in failure to thrive (FTT) and/or stunted growth, especially in children (20–23). The malabsorption syndrome observed during chronic human giardiasis is characterized by a steatorrhea type diarrhea with signs of fat- (i.e., vitamins A, K) and water-soluble (i.e., vitamin B12) vitamins deficiency (24–29). In persistent cases of giardiasis, especially in children under the age of 5, significant weight loss accompanied by a wasting protein-losing enteropathy are also present (30–33).

## *Giardia* Disrupts Tight Junction Proteins and Modulates Intestinal Barrier Integrity

Tight junction (TJ) proteins represent major components of the intercellular adhesion molecules and regulate the permeability of epithelial (i.e., intestine) and endothelial barrier functions [for a review see ref (34)]. These molecules are multi-protein complexes required for defining the structurally- and functionally-distinct basolateral and apical plasma membrane domains and are critical for the maintenance of the cell polarity and paracellular passage (34). The TJ proteins are not entirely impermeable yet the passive trans-epithelial passage of ions and small molecules occurs depending on molecule’s size and polarity (35–37). It has been clearly established that TJ proteins play critical and non-redundant roles in multiple organs. The ZO-1 or ZO-2 deficiency was embryonically lethal in mice (38, 39), whereas mice deficient for claudin 1 died shortly after the birth owing to excessive dehydration of the skin (40). While mice deficient for occludin (*Ocln^-/-^*) manifested extensive histological abnormalities as well as chronic inflammatory responses in intestinal and extra-intestinal organs (41), the genetic deletion of ZO-3 in mice did not cause any signs of developmental abnormalities (39, 42).

The integrity of the intestinal TJ proteins is essential for the epithelial impermeability against invading intestinal mucosal pathogens, confining pathogens in the lumen and preventing them to gain access to deeper mucosal layers (43, 44). The impaired TJ protein expression at the mucosal surfaces of the intestine leads to the facilitated entry and spread of enteric pathogens (45). Many enteric microbial pathogens, including enteropathogenic and enterohemorrhagic *Escherichia coli* (EPEC and EHEC, respectively) as well as *Helicobacter pylori*, secrete virulence factors that target TJ proteins in order to induce pathogenesis (43–45). The *Entamoeba histolytica* cysteine protease A5 (EhCP-A5) elicited a pro-inflammatory profile, as characterized by increased expression of IFN-γ, TNF-α, and IL-13 that correlated with impaired expression of TJ proteins claudin-2, occludin, and ZO-1 (46). The disrupted or the re-localization of TJ proteins, for example, result in an imbalanced water absorption, an increase in the intra-luminal water content in the intestine and may contribute to the diarrhea observed following the human infections with the attaching and effacing (A/E) EPEC and EHEC (45, 47).

Several lines of evidence have indicated that *Giardia* infection compromises intestinal epithelial barrier integrity in humans as well as in animal models of human giardiasis (48–52). The dysfunctional intestinal epithelial barrier during *Giardia* infection is characterized by altered expression of TJ proteins (i.e., ZO-1, claudins, occludin), increased intestinal permeability, and reduced transepithelial electrical resistance (TEER) in both murine models of giardiasis as well as in humans (51, 53). The disruption of intestinal epithelial TJ proteins is considered a milestone in the pathological changes associated with *Giardia* infection *in vitro* and *in vivo* (51, 53). The *Giardia* infection disruption of intestinal epithelial TJ proteins (i.e., ZO-1) was strain-dependent and could be reversed by using caspase-3 inhibitors or the pre-treatment with the epidermal growth factor (EGF) (50). Further clinical investigations indicated that the expression of the TJ protein, claudin 1, decreased by 71% in human subjects with giardiasis as compared with those individuals in the control group (51). It has been postulated that the disruption of the intestinal epithelial TJ proteins during *Giardia* infection leads to an increased leakage of food antigens through compromised intestinal mucosa into extra-intestinal sites and this may render infected individuals susceptible to allergic reactions commonly observed during human giardiasis (54). Notably, the translocation of commensal bacteria into extra-intestinal sites as a result of *Giardia*-induced barrier dysfunction correlated with the degradation of TJ proteins occludin and claudin-4 (55). However, it is still unclear how the bacterial translocations into extra-intestinal organs, including mesenteric lymph nodes (MLNs), would contribute to the pathogenesis of human giardiasis. It is yet to be discovered whether different strains of *G. duodenalis* would cause the differential translocations of bacteria from the intestinal lumen into extra-intestinal organs and whether this potential differential bacterial translocation could account for varied clinical symptoms associated with genetically-diverse *G. duodenalis* strains.

Several mechanisms have been proposed as to how *G. duodenalis* infection leads to loss of intestinal epithelial barrier integrity during human giardiasis as well as in murine models of human *Giardia* infection (50, 52). The attachment of *G. duodenalis* trophozoites to IECs leads to a contact-dependent alterations in the TJ protein occludin as well as the cellular redistribution of claudin-1 in fully differentiated Caco-2/TC7 cell monolayers (56). It has been suggested that alterations in the TJ proteins in the brush border (BB) occurred in a contact-dependent manner and required the lipid raft membrane of the trophozoite (56). The pre-treatment of the non-transformed human small intestinal epithelial cell line (SCBN) monolayers with EGF (57) or the myosin light chain kinase (MLCK) inhibitor (58), however, significantly prevented the attachment of the live trophozoites to the epithelial monolayers and abolished the parasite-induced disruption of the tight junctional protein ZO-1 (57). The Alerted distribution of TJ proteins, rather than changes in the expression of these proteins, has been proposed as a mechanism underlying the IEC abnormalities observed following *Giardia* infection (59).

Contact-independent mechanisms also have shown to contribute to the degradation of the TJ proteins and compromised intestinal integrity following *Giardia* infections; *Giardia* trophozoites contain a plethora of secreted molecules, including cysteine proteases (CPs), capable of degrading multiple components of the host immune system (60–62). The CPs secreted by *Giardia* trophozoites are considered emerging virulence factors that are able to degrade TJ proteins (i.e., claudin-1 and -4, occludin, E-cadherin) in IECs and are also capable of degrading chemokines expressed by parasitized IECs (63, 64). Recent evidence has suggested that giardipain-1, a cathepsin B-like enzyme, is expressed on the cell surface and flagella of *G. duodenalis* trophozoites and it can induce apoptosis in IEC-6 epithelial cell monolayers, as evidenced by membrane blebbing and the expression of phosphatidylserine on the surface of parasitized epithelial cell monolayers (52, 60, 62). Giardipain-1 was localized at the epithelial cell-cell junction interface and induced the reorganization and the degradation of occludin and claudin-1 as well as caused decreased TEER in Madin Darby Canine Kidney (MDCK) cell monolayers (52). Consistent with the proteolytic activity of giardipain-1 in degrading the TJ proteins, the pre-treatment with a selective CP inhibitor, E-64, or the siRNA targeting of giardipain-1 gene in *G. duodenalis* trophozoites led to an attenuated proteolytic activity of giardipain-1, as demonstrated by lessened epithelial insult in IEC-6 monolayers. Three major CPs localized in cytoplasm and the endoplasmic reticulum of *G. duodenalis* trophozoites were identified in a *Giardia*/epithelial cell co-culture setting and further evidence demonstrated that these CPs were capable of proteolyzing or reorganizing multiple TJ proteins, including claudins and occludin (64). Notably, *G. duodenalis* trophozoites expressing a variant surface protein, VSP9B10A, were able to induce the loss of cell-cell contact and cell detachment at the sites of the trophozoites attachment (65). The incubation of IEC-6 cell monolayers with conditioned medium obtained from *G. duodenalis* trophozoites expressing VSP9B10A/IEC-6 cell monolayers co-culture also induced cytotoxicity, whereas the monoclonal antibody blockade targeting the VSP9B10A protein expressed by trophozoites reversed those cytotoxic effects at the trophozoite/epithelial cell interface (62, 65).

Altogether, these findings demonstrate that secreted soluble proteins, including proteases, can immensely contribute to the pathogenesis of *G. duodenalis* infection *in vivo*. However, it still remains unclear how these parasite-derived CPs contribute to the immunopathology observed during giardiasis and whether vaccine candidates targeting these proteins could protect from parasite-induced immunotherapy. To further understand how TJ protein abnormalities could lead to a malabsorption syndrome (i.e., impaired absorption of electrolytes, water and disaccharidase deficiency) as well as increased intestinal permeability observed during human giardiasis, especially in younger children, further investigations are warranted (66, 67).

## *Giardia* Induces Apoptosis in Parasitized IECs

Apoptotic IECs comprised up to 1.5% of the total IECs in parasitized human duodenal biopsies following *G. duodenalis* infections, whereas 1% of the total IECs from duodenal biopsies from healthy controls were apoptotic as determined by a positive terminal transferase uridyl nick end labeling (TUNEL) staining assay (51). Apoptotic IECs, following human *G. duodenalis* infections, were characterized by chromatin condensation clustering around the nuclear periphery as well as segmentation of the nucleus (51). The *Giardia*-induced apoptosis in IECs was more evident after infection with non-host specific strains as well as following mixed infections with distinct *G. duodenalis* assemblages (68).

A wide array of mechanisms have been proposed to contribute to apoptosis induced by different genotypes of *Giardia* parasites in IECs (69). Earlier studies reported a strain-dependent induction of apoptosis in IECs following infection with a single *Giardia* assemblage or after mixed *Giardia* infections (50, 68). It was shown that the NF and S2 strains of *G. duodenalis*, but not WB or PB, were able to induce apoptosis in IECs, and these effects were abolished by pre-treating human duodenal epithelial monolayers with a caspase-3 inhibitor, Z-DEVD-FMK (50). Further studies have indicated the importance of caspases, including caspase 3 (50, 52, 70, 71) and caspase 9 (72) in mediating *Giardia*-induced apoptosis in IECs. *Giardia* infections facilitate apoptosis in IECs by the downregulation of anti-apoptotic proteins, including Bcl-2, and the up-regulation of the pro-apoptotic proteins, including Bax, suggesting a potential contribution of caspase-dependent apoptosis signaling pathways in the induction of pathogenesis during giardiasis (51, 70, 73).

The production of nitric oxide (NO), and its two major ultimate metabolites (i.e., nitrite and nitrate) by IECs represents another defensive mechanism employed against a wide range of lumen-dwelling enteric pathogens at the intestinal epithelium surface (74, 75). *Giardia* parasites interfere with the NO production by IECs through competing over local arginine availability and depriving IECs of arginine is considered a mechanism employed by *G. duodenalis* to evade NO-mediated killing of the parasite (74). Additionally, this has been suggested as a mechanism by which *G. duodenalis* induces apoptosis in parasitized IECs, since arginine deprivation is known to lead to apoptosis (74, 75).

Consistent with the observations that *G. duodenalis* strains differ in their ability to induce pathological changes at the upper intestinal epithelial surface (50, 66, 68, 76), calves infected with assemblage E neither showed increased rates of apoptotic cells nor did they exhibit any signs of villus shortening as compared with uninfected controls (77). Although the exact mechanisms underlying this discrepancy is still unclear, it is likely that the genetic loci, including triosephosphate isomerase (*tpi*), glutamate dehydrogenase (*gdh*) and β-giardin (*bg*), commonly used to assign *Giardia* parasites to specific genotypes/assemblages are not associated with virulence (2).

## *Giardia* Infection Induces Cytoskeletal Remodeling in IECs

It has been shown that parasitized IECs undergo drastic cytoskeletal remodeling following *Giardia* infection *in vitro* and *in vivo* (76). The expression and the cellular distribution of actin filaments (i.e., F-actin and alpha-actinin) as well as actin-binding proteins (i.e., villin and ezrin) are altered following *Giardia* infection, leading to compromised intestinal epithelial integrity (49, 76, 78). The co-incubation of human intestinal epithelial monolayers (i.e., SCBN and Caco2 cell lines) with live *Giardia* parasites led to local condensation of F-actin and loss of alpha-actinin in IECs as did the co-culture of monolayers with *Giardia* lysates or *Giardia* conditioned medium (49). However, Verapamil, a phenylalkylamine calcium channel blocker, did not alter F-actin reorganization suggesting an extracellular calcium independent-mechanism in the induction of cytoskeletal abnormalities following *Giardia* infection (49). Further studies demonstrated the significant contribution of host immune responses in the induction of cytoskeletal alterations following *Giardia* infection *in vivo* (76). The expression and the cellular distribution of villin and ezrin, the two crucial elements of the actin cytoskeleton of the BB of IECs, underwent major post-transcriptional changes during the clearance phase of *G. duodenalis* infection *in vivo* (76). Notably, ezrin and villin were found to be differentially regulated by immune-mediated mechanisms following *Giardia* infection; while ezrin proteolysis required CD4^+^ T cells alone, the cleavage of villin required both CD4^+^ and CD8^+^ T cell responses (76). The decreased levels of ezrin phosphorylation as well as increased levels of phosphorylated villin correlated with reduced BB disaccharidase enzymes (i.e., sucrase, maltase) activity observed during *Giardia* infection (66, 76). Altogether these observations demonstrated that both host and pathogen factors contributed to the cytoskeletal remodeling observed during giardiasis.

## *Giardia* Infection Promotes the Expression of Antimicrobial Peptides by Parasitized IECs

As the first line of defense against mucosal pathogens, IECs are equipped with a plethora of defensive mechanisms, including the ability to secret a wide array of antimicrobial peptides (AMPs) (i.e., defensins, trefoil factors) [for a review see ref (79)]. The AMPs are a diverse group of naturally occurring positively charged small molecules and are considered integral components of the innate immune system in a wide range of animals and plants (80). These proteins are crucial against invading mucosa-dwelling microbes, including bacterial, parasitic, and fungal pathogens (81, 82). Multiple AMPs, including indolicidin, a 13-residue peptide originally isolated from bovine neutrophils, as well as human defensins possessed antigiardial activity against *G. duodenalis* trophozoites *in vitro* (83).

**Figure 1:**
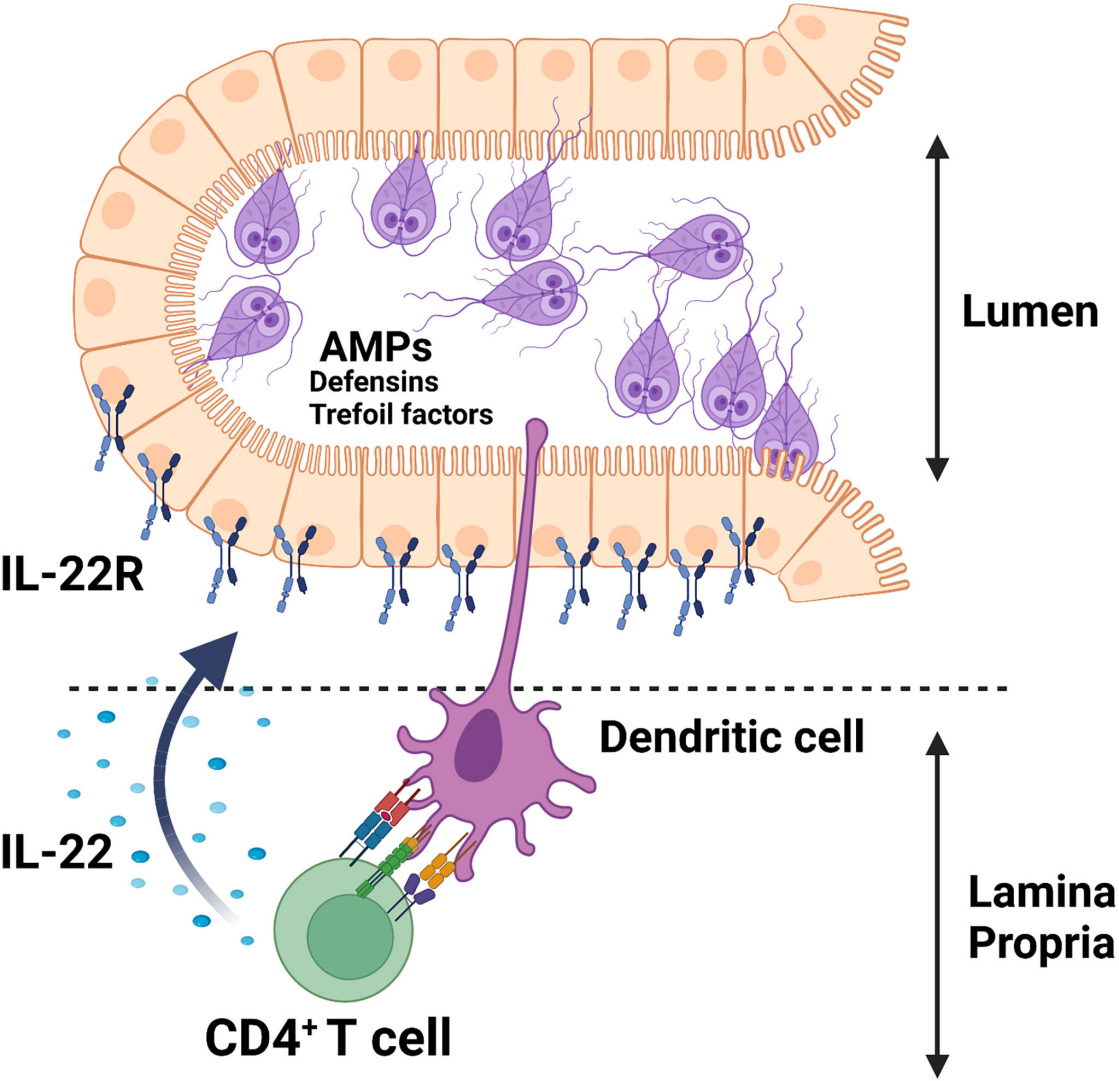
*Giardia* infection induces the secretion of AMPs (e.g., defensins, trefoil factors) in parasitized IECs likely *via* an IL-22/IL-22R-mediated mechanism.

The cytokine IL-22 is shown to promote antimicrobial responses at the mucosal surfaces of the intestine *via* the regulation of these peptide secretions through interaction with its receptor, IL-22R, which is solely expressed on non-hematopoietic cells, including epithelial cells in the intestine [for a review see ref (84)]. As depicted in **Figure 1**, we showed that *G. duodenalis* infection induces IL-22 secretion in a CD4^+^ T cell-dependent manner in a mouse model of the human *Giardia* infection (66). *Giardia* infection upregulates the expression of multiple AMPs both *in vitro* and *in vivo* (85–87). Caco-2 monolayers incubated with *G. duodenalis* trophozoites promoted the expression of human β-defensin 2 (HBD-2) and trefoil factor 3 (TFF3) (87). The upregulation of HBD-2 and TFF3 by Caco-2 monolayers was abolished by pretreatment of *G. duodenalis* with a global CP inhibitor, E-64d, or a cathepsin B CP inhibitor, Ca-074Me (87). Furthermore, *Giardia* parasite-derived proteases can cleave human defensins (i.e., α-HD6 and β-HD1) *in vitro* (64), indicating that *Giardia* parasites likely employ this strategy to evade immune-mediated killing by AMPs *in vivo*. These observations demonstrate the pivotal roles played by *Giardia*-derived proteases as contributing factors in the pathogenesis of human giardiasis and exemplify a potential strategy employed by the parasite to survive *in vivo* and suggests that these AMPs could be devised to boost host’s non-immune defense mechanisms against this pathogen.

## *Giardia* Parasites Are Closely Associated With the Intestinal Epithelium

Early studies have shown that *G. duodenalis* differentially binds to the apical surface and the basolateral membrane of murine cell lines *in vitro* (88) and trophozoite optimal growth and survival require intimate interaction with mammalian cells (89). *Giardia* trophozoites bind to small intestinal IECs with a higher affinity as compared with colonic enterocytes, consistent with the anatomical adaptation/niche in the upper portions of the small intestine (88). *Giardia* trophozoites adhere to microvilli close to the bases of the villi in the upper portions of the mouse small intestinal epithelium *in vivo* (15, 90). They also have the ability to colonize Peyer’s patches throughout the upper portions of the small intestine, but they are not found attaching to microfold cells (also known as M cells) (90). Upon infection, *Giardia* trophozoites are contained in the lumen and do not invade deeper layers of the intestine. Under certain circumstances (i.e., in immunocompromised individuals), however, *Giardia* trophozoites become invasive and are spread into intestinal mucosa extending into submucosa layer as well as extra-intestinal sites (91–93). These findings indicate the requirement of an intact immune response in order to contain the parasite within the intestinal lumen.

## Intestinal Epithelium as First Defense Layer Against *Giardia*: Role of Intestinal Mucus Layer

The mucosal surface of the intestinal tract represents a main entry point for various microbial pathogens. These microbial pathogens encounter natural innate barriers in the gut, including the mucus layer, in order to prevent potential pathogens or their immunomodulatory components/antigens to reach the underlying epithelium, a process known as non-immune exclusion (94, 95). Mucins of the human gastrointestinal tract are highly glycosylated proteins and consist of an apomucin protein backbone (100-500 kDa) joined to oligosaccharides (96). These glycoproteins are secreted by specialized epithelial cell types (i.e., goblet cells) and line the luminal surfaces of the gastrointestinal tract from the oral cavity/oropharynx to rectum (97), and act as the first line of host defense against multiple enteric microbial pathogens, including *G. duodenalis* (61). The mucin binding sites compete with those of underlying intestinal epithelium and limit attachment and the subsequent colonization of the intestinal wall by microbial pathogens (98, 99). Furthermore, the mucus layer of the intestinal tract provides a slimy and viscous physical barrier against ingested pathogens and can substantially limit their access to the underlying intestinal epithelium (100). The gut-dwelling protozoan parasites, including *G. duodenalis*, encounter natural barriers during intestinal colonization and have developed strategies to streamline this process through evading the recognition by host’s non-immune and immune mechanisms (2). The expression of mucins is upregulated following *Giardia* infection *in vitro* and *in vivo* (101–106) and can inhibit the attachment of *G. duodenalis* trophozoites *in vitro* most likely through electrostatic repulsion between the trophozoites and the underlying substratum (107). However, not all the components of the mucus possess inhibitory effects on the parasite attachment, since a non-mucin low density, protein-rich fraction of the mucus from the duodenum and ileum of humans or rabbits promoted the attachment and the survival of *G. duodenalis* trophozoites in a dose-dependent manner *in vitro* as well as protected trophozoites from being destroyed by the human milk (108–110). While lumen-dwelling protozoan parasites were drastically different in their ability to break down mucins, *G. duodenalis* produced beta-N-acetylglucosaminidase as well as detectable levels of beta-N-acetylgalactosaminidase activity, suggesting the ability of *G. duodenalis* trophozoites to efficiently break down mucins (111). These findings were further confirmed by the observations that animals infected with *G. duodenalis* exhibited a thinner mucus layer and had larger goblet cells (GCs) in greater numbers, accompanied by depleted GCs mucin stores as compared with their uninfected controls (104, 112). Consistent with the protective roles played by mucins during giardiasis, mice deficient for mucin 2 gene (*Muc2* ^-/-^) showed significantly higher trophozoite burdens in the small intestine and had impaired weight gain as compared with control animals (104). The mucus secretion is regulated by a wide range of immune (i.e., pro-inflammatory cytokines) and non-immune (i.e., diet) factors (113, 114). Diets low in fiber facilitate the overgrowth of those bacteria capable of degrading the mucus layer and promotes the subsequent *Citrobacter rodentium*-induced colitis (115). Consistently, Mongolian gerbils (*Meriones unguiculatus*) receiving a high-fiber (20%) diet were more resistant to infection with *G. duodenalis* as compared with those gerbils maintained on a diet with low fiber (5%) contents (101). The higher mucus secretion in those animals maintained on a high-fiber diet was suggested as a factor contributing to the resistance of these animals to *G. duodenalis* infection (101). These findings reveal an intricate crosstalk between *G. duodenalis* and the intestinal mucus layer at the mucosal surfaces of the small intestine. Strategies should be employed to boost non-immune innate mechanisms against intestinal microbial pathogens *via* restoring eroded mucus layer by promoting the secretion of mucus using fiber-rich diets.

## Immune Activation by *Giardia* Parasites at the Intestinal Epithelium

*Giardia* parasites are considered non-invasive to minimally invasive gut pathogens that typically reside on the epithelial surfaces of the upper portions of the small intestine (90). Yet, the adhesion of *Giardia* parasites to the intestinal epithelium triggers a strong immune response activation, as characterized by an increased influx of immune cell subtypes in the intraepithelial lymphocytes (IELs) as well as in the lamina propria lymphocytes (LPLs) of the small intestine during an early phase of the parasite’s colonization (66, 116–121). Furthermore, several lines of research have indicated that parasitized IECs secrete a wide array of chemokines and anti-giardial factors upon coming into contact with *Giardia* parasites *in vitro* and *in vivo* (19, 74, 75, 106, 122–125). The treatment of human colonic cell lines (i.e., Caco-2, HT-29) with the excretory-secretory products of *Giardia* or whole trophozoites induced the production of pro-inflammatory cytokines TNF-α, IL-1β, and IL-8 (also known as CXCL8) by these cells *in vitro* (63, 125). The degradation of CXCL8 *via G. duodenalis* cathepsin B cysteine proteases attenuates CXCL8-induced chemotaxis of human neutrophils (63, 126), indicating a potential immune evasion mechanism employed by the parasite to prevent the recruitment of neutrophils *via* a CXCL8/CXCR1/CXCR2 circuit.

## *Giardia* Infection Predisposes Infected Individuals to Disaccharidase Deficiency

Disaccharidase enzymes, including sucrase and lactase, are expressed by BB membrane and IECs in the small intestine. A decrease in the surface area of the small intestine is associated with diminished levels of disaccharidases required for the breakdown of disaccharides into absorbable monosaccharides (127). Sucrase, for example, breaks down sucrose into glucose- fructose, whereas lactase and maltase convert lactose and maltose into galactose-glucose and two glucose monomers, respectively. Undigested intact disaccharides can increase the small intestine’s osmotic pressure gradients, facilitating the secretion of large quantities of water into the intestinal lumen and leads to intestinal swelling and rapid gastrointestinal transit into the colon (128). Disaccharidase deficiency is observed following various infectious and non-infectious conditions (66, 129, 130).

**Figure 2:**
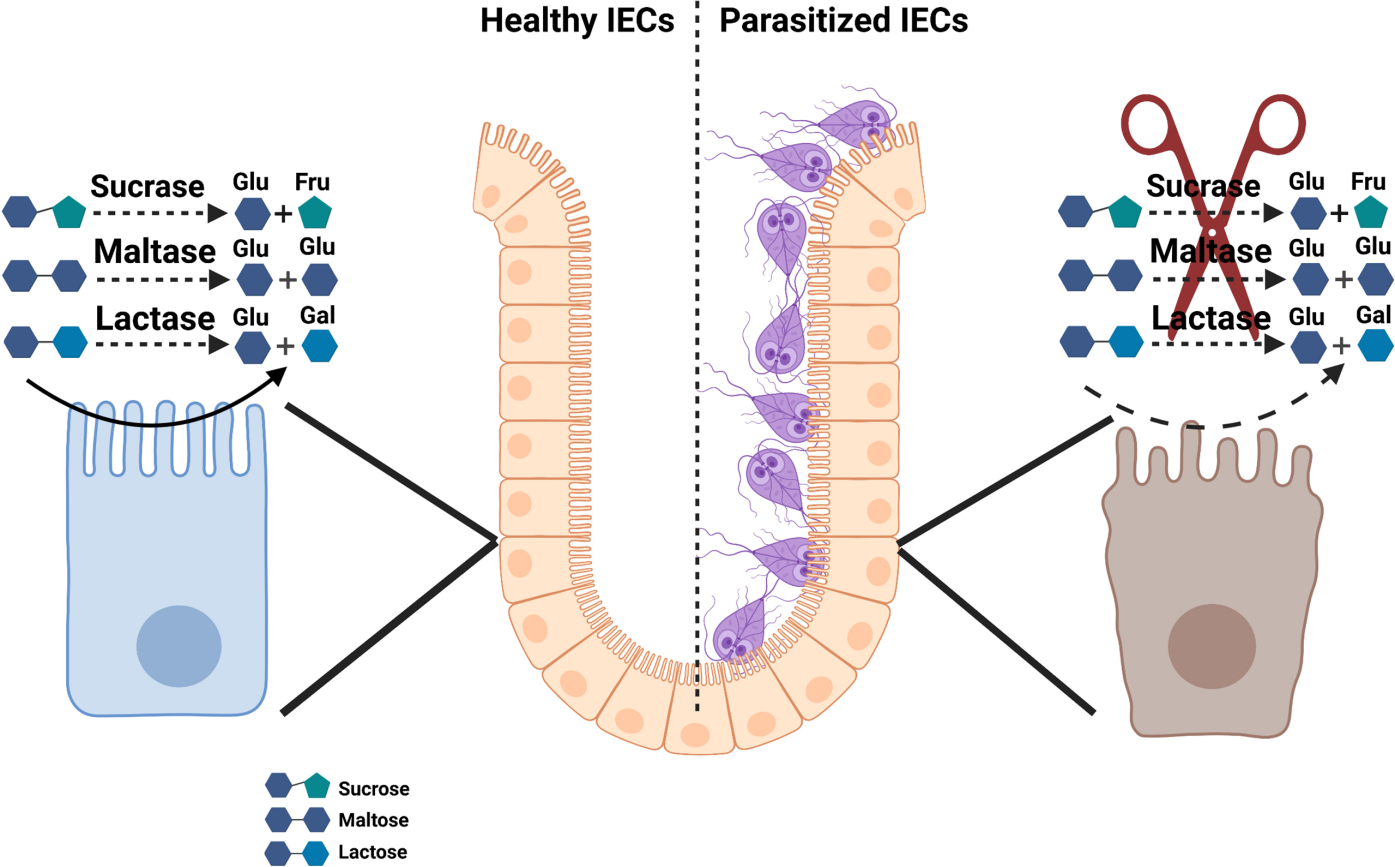
Schematic model of disaccharidase deficiency following infection with *Giardia* infection *in vivo*.

As shown in **Figure 2**, numerous studies have linked disaccharidases deficiency with *Giardia* infections in both humans and in mouse models of the human disease (57, 117, 131). Early studies demonstrated that the eradication of the parasite in human subjects infected with *G. duodenalis* led to the disappearance of clinical symptoms and the malabsorption syndrome as well as restored the villi microstructures (132). Among disaccharidases, the lactase deficiency is a common finding in *Giardia*-infected individuals (133–135), and its deficiency strongly correlates with the severity of mucosal damage in the jejunum and may persist as the lactose intolerance even after the successful chemotherapy (136).

Several mechanisms have been proposed as to how *Giardia* infection causes disaccharidase deficiency in the small intestine. Gillon et al. found a direct correlation between the impaired expression of disaccharidases and the maximal trophozoite numbers in the jejunum 2 weeks post-infection and thus proposed that the parasite’s direct effects on the jejunal BB rather than IECs immaturity, accounted for impaired levels of disaccharidase activities in a model of primary *G. muris* infection (117). In consistent with these observations, it was also proposed that the *G. duodenalis* induction of disaccharidase deficiency was a direct result of the damage to the small intestine epithelial surface rather than bacterial overgrowth, bile aids deconjugation, or immune-mediated host responses (137, 138). However, the IECs immaturity and an increase in the immature/mature IECs ratio have been proposed as a mechanism underlying disaccharidase deficiency, since immature IECs express substantially lower levels of disaccharidases as compared with mature fully-transformed IECs (76). We have demonstrated that *G. duodenalis* infection leads to a facilitated proliferation of IECs, a shift in the immature/mature IECs ratio, and an altered positional distribution/migration of IECs along the crypt-villus axis (CVA) in a primary mouse model of human infection (76). Reduced levels of ezrin phosphorylation as well as enhanced phosphorylation levels of villin correlated with diminished BB enzyme activity at the peak of *G. duodenalis* infection (76).

The primary *G. duodenalis* infection in gerbils was associated with ephemeral impairments in disaccharidase activity in the small intestine, whereas the secondary infections in these animals caused even more severe reductions in the disaccharidase levels following infection (139). Notably, the inoculum dose on the re-challenge did not correlate with reduced levels of disaccharidases activity and the presence of viable trophozoites was not required for the induction of severe enzyme deficiency during a secondary *G. duodenalis* infection (139, 140). The disaccharidase deficiency was dependent on the mouse strain and was more evident in those mice susceptible (i.e., C3H/HeN) to *G. muris* infections as compared with the resistant C57BL/6 strain (141). In an attempt to reveal the contribution of host and parasite factors, including host gender, in the induction of enzyme deficiency during *G. muris* infection, it was observed that male and female mice both had decreased enzyme activities following infection, with males exhibiting persistent reductions in enzyme activity as compared with females and a given strain of *Giardia* was associated with impaired enzyme activity, whereas as other strains were not (67, 142, 143). The significantly higher trophozoite numbers in males during a primary infection setting accounted for the gender-based differences in enzyme activity in these animals (142). The gender-biased differences in the microbiome compositions between males and females could potentially explain discrepancies observed in the levels of enzyme activity following *Giardia* infection, as the microbiome is shown to regulate disaccharidase levels through the activation of T cell subsets (121). Altogether, these findings highlighted the contribution of host factors, including host’s genetic background and gender, in the reduction of BB enzymes during giardiasis. These observations also emphasized the potential roles played by immune system during giardiasis, especially during secondary infections.

Further investigations have clearly found a more direct link between host’s immune status and disaccharidase deficiency in the small intestine following *Giardia* infection (66, 144, 145). The BB damage and the subsequent disaccharidase deficiency did not develop in the absence of T cells in nude mice following *G. muris* infection or in those mice with severe combined immunodeficiency (SCID), lacking both arms of the adaptive immunity (66, 145). The adoptive transfer of CD8^+^ T cells, but not CD4^+^ T cells, from infected mice into naïve mice led to reduced disaccharidase enzymatic activity in recipients, suggesting that CD8^+^ T cells are crucial for the induction of BB abnormalities typically observed during *Giardia* infection. As such, those mice deficient in CD8^+^ T cells (*β_2_M* ^-/-^) cleared *G. duodenalis* infection similar to their wild-type controls, whereas they did not exhibit defects in disaccharidase activity (66). Based on these findings, it is hypothetically feasible to generate protective immunity against *Giardia* infections without inducing the BB damage, including disaccharidase deficiency.

## Conclusion

The mucosal surface of the intestinal tract represents a major interface for host-microbe interaction and the main entry route for many microbial pathogens, including *Giardia* parasites. Intestinal epithelial cells are integral components of an intricate network of immune and non-immune players responsible for the maintenance of the intestinal homeostasis. As a major mucosal surface interfacing between the “self” and the “non-self”, the intestinal epithelium participates in host defense against a wide range of lumen-dwelling intestinal pathogens by secreting multiple immune mediators with direct anti-microbial properties. The *Giardia* attachment to the intestinal epithelium is considered an essential step towards the parasite colonization and the subsequent induction of pathological changes observed during human giardiasis. However, the mechanisms by which *Giardia* parasites intimately associate with the intestinal epithelium are not fully understood. To this end, a comprehensive understanding of the crosstalk between the intestinal epithelial layer and *Giardia* parasites will provide insights into the roles contributed by host and parasite factors in the development of immunopathology during human infections and will further provide mechanisms to harness dysregulated immune responses in patients with giardiasis and may offer novel therapeutic targets for the treatment of these patients.

## Author Contributions

Conceptualization, writing—original draft, reviewing, and editing: SS-M.

## Funding

Research in the Laboratory of Mucosal Immunology is supported by a startup fund (20344-8015) from the Department of Biomedical Sciences, School of Medicine and Health Sciences, University of North Dakota (to SS-M), a Dean’s Meritorious Pilot Grant, School of Medicine and Health Sciences, University of North Dakota (to SS-M), and by NIH/NIGMSP20GM113123 (to SS-M).

## Conflict of Interest

The author declares that the research was conducted in the absence of any commercial or financial relationships that could be construed as a potential conflict of interest.

## Publisher’s Note

All claims expressed in this article are solely those of the authors and do not necessarily represent those of their affiliated organizations, or those of the publisher, the editors and the reviewers. Any product that may be evaluated in this article, or claim that may be made by its manufacturer, is not guaranteed or endorsed by the publisher.

## Abbreviations

AMPs, antimicrobial peptides; A/E, attaching and effacing; BB, brush border; CPs, cysteine proteases; CVA, crypt-villus axis; EGF, epidermal growth factor; EHEC, enterohemorrhagic *Escherichia coli*; EPEC, enteropathogenic *Escherichia coli*; FTT, failure to thrive; GC, goblet cell; HBD-2, human β-defensin 2; IEC, intestinal epithelial cell; IELs, intraepithelial lymphocytes; LPLs, lamina propria lymphocytes; MDCK, Madin Darby canine kidney; MLCK, myosin light chain kinase; MLNs, mesenteric lymph nodes; NO, nitric oxide; SCID, severe combined immunodeficiency; TEER, transepithelial electrical resistance; TFF3, trefoil factors 3; TJ, tight junction; TUNEL, terminal transferase uridyl nick end labeling; ZO-1, zonula occludens-1
